# Latest Perspectives on Alzheimer’s Disease Treatment: The Role of Blood-Brain Barrier and Antioxidant-Based Drug Delivery Systems

**DOI:** 10.3390/molecules29174056

**Published:** 2024-08-27

**Authors:** Bianca Sânziana Daraban, Andrei Sabin Popa, Miruna S. Stan

**Affiliations:** 1 Department of Biochemistry and Molecular Biology, Faculty of Biology, University of Bucharest, 91-95 Splaiul Independentei, 050095 Bucharest, Romania; daraban.bianca-sanziana22@s.bio.unibuc.ro (B.S.D.); andrei.popa2@s.unibuc.ro (A.S.P.); 2 Research Institute of the University of Bucharest—ICUB, University of Bucharest, 050095 Bucharest, Romania

**Keywords:** antioxidant molecules, Alzheimer’s disease, blood-brain barrier

## Abstract

There has been a growing interest recently in exploring the role of the blood-brain barrier (BBB) in the treatment of Alzheimer’s disease (AD), a neurodegenerative disorder characterized by cognitive decline and memory loss that affects millions of people worldwide. Research has shown that the BBB plays a crucial role in regulating the entry of therapeutics into the brain. Also, the potential benefits of using antioxidant molecules for drug delivery were highlighted in Alzheimer’s treatment to enhance the therapeutic efficacy and reduce oxidative stress in affected patients. Antioxidant-based nanomedicine shows promise for treating AD by effectively crossing the BBB and targeting neuroinflammation, potentially slowing disease progression and improving cognitive function. Therefore, new drug delivery systems are being developed to overcome the BBB and improve the delivery of therapeutics to the brain, ultimately improving treatment outcomes for AD patients. In this context, the present review provides an in-depth analysis of recent advancements in AD treatment strategies, such as silica nanoparticles loaded with curcumin, selenium nanoparticles loaded with resveratrol, and many others, focusing on the critical role of the BBB and the use of antioxidant-based drug delivery systems.

## 1. Introduction

Alzheimer’s disease (AD), a debilitating neurodegenerative condition, continues to afflict a growing number of individuals globally, posing a significant public health concern. Nowadays, this disease affects 6.7 million older Americans, with great potential of rising to 13.8 million by 2060 without medical breakthroughs; the reported deaths from AD increased by more than 145% between 2000 and 2019 [1]. Beyond the traditional focus on amyloid-beta deposition and tau hyperphosphorylation, the latest evidence suggests that factors such as insulin resistance in the brain, disruption of the Wnt-β catenin pathway, and oxidative stress also play crucial roles in the progression of the disease [2]. To address these multifaceted challenges, researchers have explored the potential of nanomedicine-based approaches [3].

The blood-brain barrier (BBB), essential for protecting the brain from harmful agents, also poses a significant challenge in delivering therapeutic agents to the affected regions. Strategies that can effectively navigate this barrier and target the specific pathological mechanisms of Alzheimer’s disease are crucial for developing more effective treatments.

In this context, the present review highlights the underlying mechanisms of AD and the altered BBB function in this disease, including increased permeability, impaired efflux mechanisms, and disrupted nutrient transport. These alterations contribute to the accumulation of toxic substances in the brain, including amyloid-beta plaques and tau tangles, which are hallmarks of AD.

Furthermore, this review discusses promising advancements in antioxidant-based drug delivery systems by using nanoparticles and liposomes as carriers, which include optimizing drug loading, targeting specificity, and avoiding systemic toxicity. Antioxidants, such as curcumin, resveratrol, and quercetin [4], have neuroprotective properties and can help mitigate oxidative stress, a major contributing factor to AD progression. By incorporating antioxidants into drug delivery systems, researchers aim to improve the BBB permeability of drugs and enhance their therapeutic efficacy.

Nanomedicine-based approaches offer innovative solutions to the complex challenges of AD by aiding targeted drug delivery through the BBB, which helps reduce oxidative stress and modulate neuroinflammation. These approaches allow precise therapeutic action at the cellular level, addressing various AD-related factors such as amyloid-beta accumulation, neurofibrillary tangles formation, and neuronal degeneration, improving treatment outcomes and slowing disease progression. These findings open up new possibilities for targeted and efficient drug delivery systems for Alzheimer’s treatment, offering hope for more effective therapies with fewer side effects.

## 2. Molecular Mechanism of Alzheimer’s Disease

AD is characterized by progressive cognitive decline and memory loss, significantly impairing daily functioning and quality of life. As the global population ages, the prevalence of AD is expected to rise, posing a substantial public health challenge. The latest data suggest that by 2050, dementia prevalence will double in Europe and will triple globally [5]. AD is a multifactorial dysfunction influenced by a variety of factors, both genetic and non-genetic. Key factors include environmental influences, age, lifestyle, and biological factors such as apolipoprotein E (ApoE) variants, insomnia, insulin resistance, and alterations in gut microbiota [6].

Apprehending the intricate molecular pathways involved in AD pathogenesis is essential for establishing therapeutic targets and developing innovative therapies. The two primary pathological hallmarks of AD are amyloid-β plaque formation (Aβ) and neurofibrillary tangles (NFTs), consisting of hyperphosphorylated tau [7]. As AD progresses, these aggregates disrupt neuronal function and lead to synaptic loss and neurodegeneration.

This chapter aims to review the current understanding of the molecular mechanisms underlying Alzheimer’s disease, with a focus on the roles of Aβ and tau, genetic and metabolic factors, and neuroinflammation.

### 2.1. Amyloid-Beta and Tau Dynamics

Aβ formation is based on a crucial cellular mechanism involving the proteolytic processing of amyloid precursor protein (APP), highly expressed in neurons. In the healthy brain, Aβ has several physiological roles, including neurite growth regulation, axonal guidance, neuroprotection, and early development of the nervous system [8]. Additionally, some aspects of APP function derive from its cleavage products, such as soluble amyloid precursor proteins (sAPP) α and β. The function of (sAPP) α has been extensively characterized, playing a significant role in neuronal plasticity, neuronal stem cell proliferation, and survival, also protecting against Aβ induced toxicity [9].

APP can be cleaved in two distinct pathways: non-amyloidogenic and amyloidogenic. In the non-amyloidogenic route, APP is firstly processed by α-secretase, followed by γ-secretase, which cuts within the Aβ domain, preventing Aβ formation. The amyloidogenic pathway is present in AD patients, where APP is sequentially cleaved by β-secretase and then γ-secretase, resulting in the extracellular release of Aβ fragments of varying lengths, primarily consisting of 40 (Aβ40) or 42 (Aβ42) amino acids [10]. These fragments can aggregate, consequently forming insoluble amyloid fibrils [11] that are the foundation of synaptic and neuronal dysfunction in AD [6].

The human tau protein, encoded by the MAPT gene on chromosome 17, plays a crucial role in stabilizing axonal microtubules [12]. Tau is abundantly expressed in neurons in the mammalian brain and is mainly found in axons, where it is essential for regulating axonal transport. Tau is also found in oligodendrocytes and astrocytes [13], where it plays a role in processes such as outgrowth, myelination, and potentially other physiological functions. Furthermore, tau is known to regulate iron export and insulin signaling [14].

Tau distribution in neurons and its involvement in synaptic function are controlled by post-translational modifications, such as phosphorylation, methylation, sumoylation, nitration, glycosylation, and acetylation [6]. Among these various modifications, of utmost importance is phosphorylation since this process is involved in a class of neurodegenerative disorders called tauopathies, where AD is included [15]. Tauopathies are characterized by the presence of abnormal neuronal and glial inclusions of tau protein, which give rise to hyperphosphorylated insoluble aggregates [15]. During AD pathogenesis, the main event is hyperphosphorylation of tau. In this case, the affinity of tau for tubulin decreases, and, as a consequence, tau will accumulate in the cytosol and form insoluble structures known as neurofibrillary tangles [7].

One key concept for the propagation of tau in the human brain is the prion-like model, where abnormal forms of tau can easily spread from one cell to another, disseminating the pathology from affected areas to healthy regions, with a similar mechanism to prion diseases [16]. Multiple groups of scientists support this theory because it has been observed that introducing extracts containing tauopathy into normal brains can induce this pathology in the recipient subjects, with the propagation starting from the injection site [17,18].

Completely understanding the underlying mechanisms of AD involves focusing on the intricate interplay between Aβ and tau proteins rather than examining them separately. Their interaction involves intermediate molecules like kinases, as proposed by Zheng et al. [19] in 2002. The relationship between Aβ and tau is multifaceted: while Aβ promotes tau pathology by triggering tau hyperphosphorylation, hyperphosphorylated tau, in turn, contributes to neuronal toxicity. On the other hand, tau also plays a role in Aβ toxicity, and its presence is crucial for the toxic effects of Aβ [20]. Aβ and tau collaborate to target cellular processes and organelles, potentially exacerbating each other’s harmful effects. Furthermore, tau oligomers, the precursors to NFT, are toxic [21], and their aggregation is facilitated by Aβ-activated CDK-5 and GSK-3β [22].

### 2.2. Chronic Inflammation in Alzheimer’s Disease

Even though neuroinflammation is not typically associated with the initiation of AD, it can significantly worsen the disease by aggravating amyloid-beta (Aβ) and tau pathologies. The release of inflammatory cytokines by immune cells further activates the immune system and enhances inflammatory responses [23]. AD patients have been found to exhibit persistent and excessive activation of immune cells, potentially leading to the build-up of Aβ and tau, loss of synapses, disruption of the blood-brain barrier (BBB), and neurodegeneration [24].

Elevated cytokine levels can impact other processes related to AD as well. For example, interleukin-18 (IL-18) increases the levels of enzymes involved in tau hyperphosphorylation [25]. The activation of the Cdk5 pathway also leads to Golgi fragmentation, as well as neuronal and mitochondrial fragmentation [26]. Lately, it has been discovered that viral infections, particularly herpes viruses (HSV1 and HSV2), are also associated with AD. These viruses can trigger Aβ aggregation, and their DNA is commonly found in Aβ plaques. Reactivation of HSV1 is associated with tau hyperphosphorylation and possibly tau propagation [12].

Another important factor contributing to the neuroinflammation observed in AD pathology is the activation of the NLRP3 inflammasome [27]. Misfolded protein conglomerates, such as Aβ plaques, are believed to be involved in the activation of NLRP3 in microglia [28]. This activation results in the release of active IL-1β and caspase-1, and levels of these molecules have been shown to be increased in both AD patients and animal models [29]. In AD, microglia are drawn to neuritic plaques to consume aggregated Aβ, triggering NLRP3 inflammasome activation, resulting in the release of proinflammatory cytokines (IL-1β and IL-18) and potentially neurotoxic factors, which can worsen the effects of Aβ and exacerbate AD pathology. It has also been observed that in transgenic APP/PS1 mice, Aβ activates the NLRP3 inflammasome in microglia, causing them to adopt a pro-inflammatory M1 phenotype characterized by high expression of caspase-1 and IL-1β, a state associated with response to cellular damage or harmful stimuli, a prolonged activation leading to increased hippocampal and cortical Aβ deposition, neuronal loss, and cognitive impairment [29].

### 2.3. Impact of Oxidative Stress in Alzheimer’s Disease

One of the main aspects of AD pathology is the imbalance between the synthesis and degradation of Aβ through self-regulatory pathways, resulting in the overproduction of Aβ in brain tissue [30]. Aβ itself can induce oxidative stress, and oxidative stress can also lead to increased production of Aβ. Neurons are known to be especially susceptible to oxidative stress due to their low antioxidant content and membranes rich in polyunsaturated fatty acids [31].

Aβ plaques disrupt normal mitochondrial activity, causing dysfunction and leading to oxidative stress [32]. Aβ reduces the efficiency of electron transfer, resulting in increased production of reactive oxygen species (ROS), impacting different mitochondrial components [33]. Additionally, Aβ has been found to inhibit mitochondrial superoxide dismutase (MnSOD), a critical enzyme for neutralizing superoxide anions and safeguarding against peroxidative damage [34].

Elevated concentrations of ROS encourage tau phosphorylation, destabilizing microtubules, which often leads to decreased synapse function [34]. Furthermore, increased oxidative stress causes an imbalance between pro- and anti-apoptotic processes, leading to apoptosis and subsequent neurodegeneration [35].

### 2.4. Role of Insulin Resistance in Alzheimer’s Disease

Impaired brain insulin signaling, known as “brain insulin resistance”, promotes cognitive dysfunction and accelerates AD progression [36]. Aβ has been found to compete with insulin for binding to insulin receptors (IR), reducing insulin binding affinity and resulting in insulin resistance, which exacerbates existing AD pathology [37]. The increased presence of these Aβ oligomers in AD patients inhibits insulin signaling pathways, leading to neuroinflammation and neurodegeneration through elevated Aβ concentrations and GSK3β-dependent hyperphosphorylation of tau [38].

Insulin plays a crucial role in cellular survival, metabolic activities, and neuronal plasticity, having neuroprotective effects and inhibiting the formation and aggregation of Aβ plaques and NFT in the cortex and hippocampus, the primary pathological features of AD, thus improving memory and cognitive functions [37]. Therefore, insulin resistance increases detrimental factors such as oxidative stress, cytokine production, and apoptosis [36].

### 2.5. Association between Alzheimer’s Disease and Gut Microbiota

The gut microbiota and the brain communicate through various pathways, such as producing pro- and anti-inflammatory cytokines, short-chain fatty acids (SCFAs), branched-chain amino acids, and neurotransmitters, influencing processes such as neurotransmitter production and metabolism, and modulating the vagus nerve and enteric nervous system [39]. These mechanisms allow the gut microbiota to play an active role in the development of various brain disorders, including AD. Studies have shown that germ-free (GF) mice have decreased levels of serotonin (5-HT) in their blood compared to mice with normal gut microbiota [40]. Since serotonin can reduce amyloid-beta (Aβ) plaque formation and the risk of AD, it is suggested that GF conditions may modulate Aβ pathology through alterations in serotonin [41].

**Figure 1:**
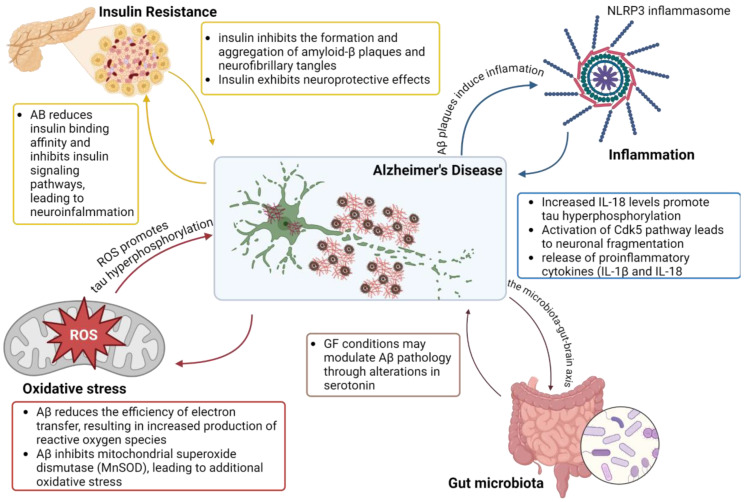
Schematic representation of mechanisms involved in Alzheimer’s Disease pathology. Created with BioRender.com (accessed on 14 August 2024).

To conclude, the molecular mechanisms underlying AD (Figure 1) predominantly revolve around the production and aggregation of Aβ peptides and the formation of NFT composed of hyperphosphorylated tau protein; insulin resistance and the brain-gut axis are critical parameters in the complexity of this disease.

## 3. The Role of Blood-Brain-Barrier in the Pathophysiology of Alzheimer’s Disease

In this chapter, we will explore the role of BBB in AD. We will examine how BBB dysfunction may influence the accumulation of Aβ and tau protein, contributing to disease progression.

### 3.1. Physiology of the BBB

**Figure 2:**
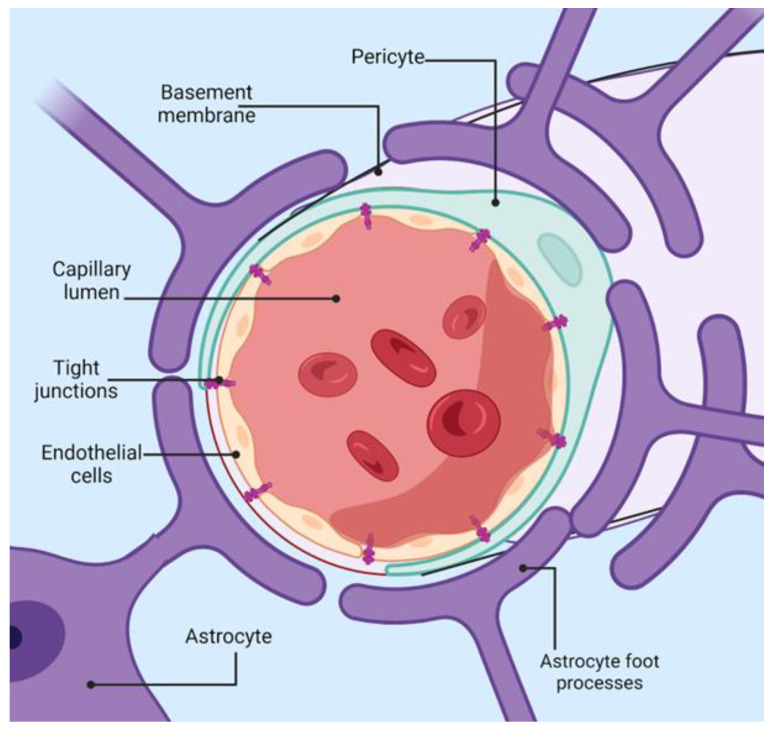
Schematic representation of blood-brain-barrier architecture. Created with BioRender.com (accessed on 14 August 2024).

The BBB represents a crucial protective mechanism that maintains the brain’s homeostasis, being a semi-permeable barrier that, together with an ensemble of transporters, receptors, efflux pumps, and more cellular components, controls the entrance and expulsion of molecules to the brain [42]. The main elements of the BBB are pericytes, astrocytes, and microvascular endothelial cells, as shown in Figure 2. At the same time, microglia and neurons are also taken into account as members of the neurovascular unit (NVU), an extended term that emphasizes the cross-talk between the periphery and the central nervous system (CNS) across the BBB [43].

Endothelial cells (EC) are the primary anatomical structure that lines the cerebral blood vessels and interacts with various cellular types from the CNS. These cells are distinct from peripheral endothelial cells in both form and function. They have a flattened appearance and are linked by tight junctions (TJ) and adherens junctions (AJ). Moreover, they have separate luminal and abluminal membrane compartments [44]. Notably, these cells lack fenestrations, which hampers the free diffusion and exchange of molecules across the BBB. EC from the blood-brain barrier has an increased amount of mitochondria, indicating the need for more energy for transport across the BBB, unlike other cellular barriers. Brain microvascular endothelial cells possess a net negative surface charge, which means they do not transport negatively charged compounds. They also contain specialized surface transporters that control the movement of specific substances. These unique characteristics of EC enable rapid oxygen diffusion from the blood to the brain and carbon dioxide diffusion in the opposite direction, playing a vital role in brain metabolism and pH regulation [45].

Pericytes (PCs) share a basement membrane with endothelial cells and directly interact with the endothelium through molecules such as N-cadherin and connexins [46]. Their primary function is to uphold the BBB integrity, aiding in the formation of new blood vessels and the stability of small blood vessels. PCs have contractile properties similar to smooth muscle cells, enabling them to control the diameter of capillaries and the blood flow in the brain [47]. Co-cultures of endothelial cells and pericytes are more resilient to cell death than isolated endothelial cells, underscoring the support provided by pericytes for the structural integrity and formation of the BBB [46]. Astrocytes, some of the most abundant cells in the CNS, are involved in various tasks, such as maintaining ionic homeostasis and neurotransmitter uptake [48]. Their primary role in the BBB is to uphold the function of EC.

The BBB regulates the levels of neurotransmitters and controls the entry of large molecules from the bloodstream into the brain. Additionally, it serves as a protective barrier, guarding the brain against toxins [45]. To maintain a stable environment (homeostasis), the BBB contains specific ion channels and transporters that ensure optimal potassium, sodium, and calcium levels. The transfer of neurotransmitters from the brain to the blood mainly relies on Na^+^-coupled and Na^+^-independent amino acid transporters. The BBB restricts the entry of specific amino acids, including glutamate and glycine, while allowing the exit of many other essential amino acids [49].

If the BBB is damaged, large serum proteins can leak into the brain, leading to severe consequences. For instance, the leakage of plasma proteins like albumin, prothrombin, and plasminogen can harm nervous tissue, causing cellular activation that may lead to apoptosis [45].

### 3.2. Transport Mechanisms across the Blood-Brain Barrier

**Figure 3:**
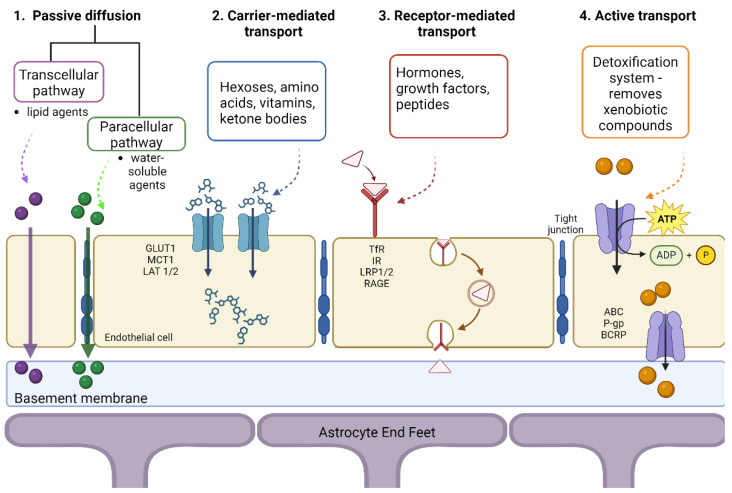
Mechanisms of transport across the blood-brain-barrier. Created with BioRender.com (accessed on 14 August 2024).

Tight junctions play a significant role in restricting the passage of molecules from one compartment to another at the BBB level, as presented in Figure 3. Hydrophilic molecules must cross the transcellular endothelial wall to reach the brain or blood. There are multiple ways of transport: passive diffusion, active transport, carrier-mediated, and receptor-mediated [50].

#### 3.2.1. Carrier-Mediated Transport

The carrier-mediated transport process uses nutrients such as hexoses (galactose, glucose, mannose), monocarboxylic acids, amino acids, nucleosides, amines, and vitamins [51]. Generally, the concentration gradient aids in transporting these nutrients from the blood to the brain, and this process is regulated by the metabolic needs of the brain and the substrate concentration present in the plasma. Because glucose represents the main energy source for the cerebrum, the GLUT1 transporter plays a crucial role, being exclusively expressed on the BBB. Its uneven distribution in the membrane allows for homeostatic control of glucose influx from the blood to the brain, preventing excessive accumulation of glucose in the brain [50]. Another transporter found in the BBB is MCT1 since another energy source for the brain is ketone bodies, R-β-hydroxybutyrate, and acetoacetate, which are monocarboxylate compounds. For amino acid transport, two transporter systems have been described: (i) the L1 system is sodium-independent and facilitates the transport of neutral amino acids with high molecular mass, such as valine, leucine, isoleucine, and (ii) the γ+ system, involved in transporting cationic amino acids, which may be essential for the adult brain (lysine) or non-essential (ornithine, arginine) [52].

Another transport system is sodium-dependent, dealing with excitatory amino acids (EAA), such as glutamate and aspartate, which is also involved in maintaining a low level of glutamate in the brain. An increased amount of glutamate in the brain can lead to brain damage and stroke, as well as neurodegenerative processes, epilepsy, Alzheimer’s, and Huntington’s disease [53].

#### 3.2.2. Receptor-Mediated Transport

Due to the presence of peptide bonds, larger peptides and proteins have limited passage through the BBB since they cannot use carriers for transport. In the case of hormones, growth factors, neuroactive peptides, and other molecules, transport is mediated by different receptors, allowing these solutes to enter the CNS through transcytosis [50]. These macromolecules bind to specific receptors found on cell surfaces, cluster together, form a vesicle, and are internalized into endothelial cells and released at the opposite side of the cells. This process involves various receptors, such as insulin receptor (IR), low-density lipoprotein receptor (LDLR), transferrin receptor (TfR), nicotinic acetylcholine receptors (nAChRs), and more [54]. These transport systems have been used as a target for the delivery of drugs to the brain through a technique called the “Trojan horse”, in which the therapeutic agent is “hidden” inside a carrier that is recognized and accepted by the target cells and is released upon targeted delivery. In this sense, growth factors or molecules that do not usually cross the BBB can be conjugated to monoclonal antibodies to barrier receptors (insulin, transferrin), the antibodies acting as surrogate ligands to transport drugs across the BBB [55].

#### 3.2.3. Active Transport 

The main components involved in active transport through the BBB are ATP-binding cassette (ABC) proteins located on the luminal endothelial side of the barrier. The primary efflux transporters expressed at the BBB level are P-glycoprotein (P-gp), also known as Multidrug Resistance Protein 1 (MRP1, ABCB1), and Breast Cancer Resistance Protein (BCRP, ABCG2) [56]. P-gp and BCRP are highly expressed in the BBB and are responsible for moving substances from the endothelium to the blood.

#### 3.2.4. Passive Diffusion

Passive diffusion is a process in which energy is not required, being driven by the concentration gradient of the unbound compound on both sides of the endothelial cell membrane. Tight junctions allow passive diffusion only for lipid-soluble drugs with a molecular weight lower than 400–600 Da [42]. Generally, molecules with high lipophilicity and small molecular size can passively diffuse across the BBB in the direction of the concentration gradient without needing energy input [57].

Transport can occur between the EC (paracellular) or through the cells (transcellular). The paracellular pathway is the main route, blocked by the tight junctions of ECs, restricting ions, polar solutes, and most macromolecules. However, tight junctions are imperfect, so small and soluble substances can cross through the paracellular pathway. The transcellular path is the preferred route for carriers to enter and transport therapeutic compounds. The molecules can partition into the cellular membranes from the top to the bottom-lateral side through carrier-mediated and receptor-mediated transcytosis [3]. In addition, lipophilic carriers and other known carrier systems like cationic amino acids are mainly transported via the transcellular pathway.

### 3.3. Disruption of Blood-Brain Barrier in Alzheimer’s Disease

**Figure 4:**
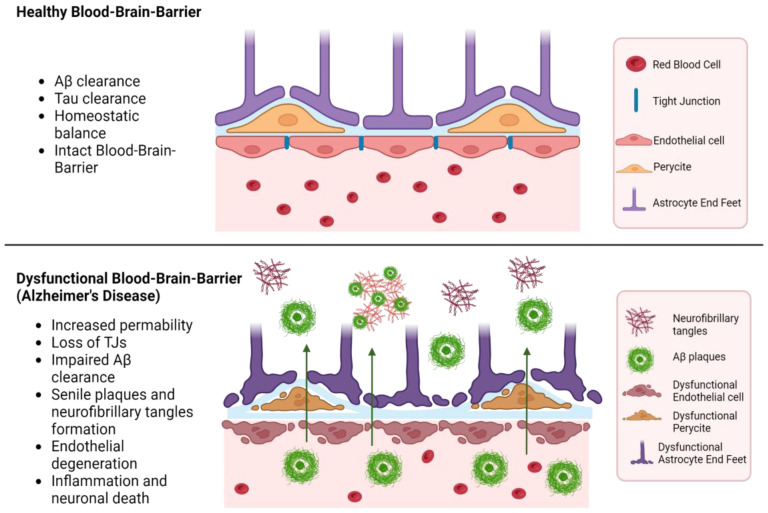
Dysfunctional blood-brain barrier in Alzheimer’s disease. Created with BioRender.com (accessed on 14 August 2024).

The progression of AD involves significant disruption of the BBB, primarily due to vascular degradation, altered molecular transport, angiogenesis issues, and inflammatory responses, as shown in Figure 4. These dysfunctions lead to continuous neural disturbances [58]. Early in AD, the selective nature of the BBB deteriorates, allowing uncontrolled entry of molecules, which contributes to disease pathogenesis [59].

#### 3.3.1. Permeability of the Blood-Brain Barrier in Alzheimer’s Disease

A key mechanism leading to BBB dysfunction is the reduction in pericyte coverage and the increase of transcytosis, resulting in a greater permeability, allowing neurotoxic proteins to enter the brain. This triggers a neuroinflammatory response driven by activated glial cells and microglia, further exacerbating BBB disruption by releasing pro-inflammatory mediators. Genetic factors, such as the presence of the ApoEε4 allele, also play a significant role in BBB breakdown [60]. Apolipoprotein E (ApoE) is involved in lipid metabolism, and the ε4 allele is associated with an increased risk of AD, exacerbating amyloid-β deposition and contributing to neuroinflammation and dysfunction of the medial temporal lobe. The receptor for advanced glycation end products (RAGE) facilitates the transport of Aβ into the brain, while the low-density lipoprotein receptor-related protein (LRP-1) contributes to its clearance. In AD, there is a shift in the distribution of RAGE and LRP-1 receptors, indicating a change in the proportion of accumulated amyloid due to BBB dysfunction [61].

#### 3.3.2. Neuroinflammation in Alzheimer’s Disease

Neuroinflammation critically contributes to BBB disruption during AD. The interaction between amyloid-beta and endothelial cells can decrease the expression of tight junction proteins. The interaction between Aβ42 and RAGE regulates tight junctions through the calcium-calcineurin signaling pathway. Structural deterioration of tight junction proteins, including claudin-5 and occludin, extensively contributes to BBB breakdown [59]. This disruption can cause various impairments of the neurovascular unit components, further expanding AD pathology. Oxidative stress during BBB neuroinflammation is characterized by increased ROS production, endothelial cell dysfunction, activation of matrix metalloproteinases (MMPs), and disruption of redox-sensitive transcription factors. In cases of peroxidation-induced activation, endothelial cells increase BBB permeability, mediated by inflammatory mediators and immune cell adhesion molecules [59].

This chronic inflammation disrupts the BBB, leading to the infiltration of peripheral immune cells and additional neuroinflammatory processes. During AD, the gene expression related to microglial homeostasis is downregulated, while those involved in the inflammatory response are upregulated [62]. Consequently, the BBB becomes more permeable, allowing neurotoxins to enter the brain, thus exacerbating neurodegeneration.

#### 3.3.3. Amyloid-β Deposition and Blood-Brain Barrier Dysfunction

The highly neurotoxic peptide Aβ42 accumulates in the brain, leading to inflammation and oxidative stress, which increase BBB permeability. Furthermore, Aβ interacts with components of the neurovascular unit, impairing their function and integrity. Aβ oligomers induce pericyte-mediated endothelial dysfunction, resulting in further BBB breakdown, marked by decreased expression of tight junction proteins, claudin-5, and occluding [63].

A critical factor in the progression of AD is the diminished capacity of the BBB to handle the influx/efflux of Aβ, the key transporters being LRP1 and P-gp. Normally, these proteins help clear Aβ deposits from the brain. However, during AD, the expression of these transporter proteins is significantly affected. These changes lead to an increased influx of Aβ into the brain facilitated by RAGE receptors [64].

## 4. Drug Delivery Systems with Antioxidant Molecules for Targeting the Treatment of Alzheimer’s Disease

### 4.1. Impact of Natural Antioxidant Molecules in AD Treatment

#### 4.1.1. Curcumin

Curcumin has the ability to bind to senile plaques with great affinity, which results in a drastic reduction in Aβ levels, the mechanism involving the decomposition of the β-sheet structure of protein aggregates at doses of 50 mg/kg/day. The Akt phosphorylation can be activated by curcumin, which also deactivates GSK-3β, reducing Aβ production and plaque deposition [65]. Another target is the nuclear factor kappa B (NF-κB), which is found at higher levels in AD patients. Also, curcumin downregulates the cytokines involved in AD, thereby reducing neuroinflammation [65].

Due to the poor bioavailability of curcumin, which is determined by its low aqueous solubility and poor permeability within the BBB, a series of drug-delivery systems and biotechnologies have been developed for efficient administration. Some of these approaches include isomerization, the use of liposomes, polymeric nanoparticles, and phospholipid complexes. One of the most used technologies involves poly (lactide-co-glycolide) (PLGA) and polyethylene glycol (PEG) [66]. Mathew and colleagues obtained promising results when exposing PLGA-curcumin nanoparticles tagged with Tet-1 peptide to amyloid protein aggregates [67]. Tet-1 peptide is a 12-amino acid peptide responsible for retrograde delivery in neuronal cells. The anti-amyloid activity was time-dependent, with the nanoparticles decomposing the aggregates slowly. In the first 12 h, PLGA-curcumin nanoparticles attached and reduced the size of these aggregates, continuing with significant breakdown in the first 24 h and resulting in smaller plaques after significant disaggregation by 48 h [67]. Huo and his team conducted an experiment using Se/Cur-PLGA nanospheres administered intravenously into transgenic 5XFAD mice with AD, demonstrating an enhanced efficiency of penetrating the BBB, being mainly located on the amyloid plaques after completely crossing the BBB [68].

To overcome the limitations of free curcumin, encapsulation in biodegradable nanoparticles, such as polymeric micelles, is the preferred strategy. This encapsulation significantly enhances the solubility of curcumin in the biological medium and protects the molecule from premature degradation, either enzymatic or oxidative. Furthermore, nanoparticles enhance the controlled release of curcumin, ensuring its concentration in target tissues, such as the brain. Subsequently, nanoparticles can be modified to facilitate transport across BBB, resulting in higher delivery of curcumin to cerebral tissues affected by AD [69].

Another characteristic of curcumin delivery is the conjugation of nanoparticles with the B6 peptide, which is responsible for the administration of curcumin at the cerebral level and its passage across the BBB [66].

While initial findings regarding curcumin were promising, ongoing clinical trials have shown that despite curcumin being well tolerated, there is no clinical evidence of its cognitive enhancement in AD, as systematically reviewed by Voulgaropoulou et al. [70], primarily due to its low bioavailability.

#### 4.1.2. Quercetin

Quercetin is able to downregulate pro-inflammatory cytokines such as NF-κB and iNOS, reducing the neuroinflammatory response and improving learning, memory, and overall cognitive functions. One of the most significant mechanisms of quercetin’s efficacy in AD is the inhibition of acetylcholinesterase (AChE), which prevents the degradation of acetylcholine, resulting in reduced Aβ aggregate production. AChE is responsible for the degradation of acetylcholine, and inhibiting it leads to higher levels of acetylcholine, thus improving the symptoms of mild to moderate AD. Quercetin inhibits AChE by binding with the enzyme, resulting in increased levels of acetylcholine in the synaptic cleft [71]. Quercetin can destabilize fibril structures through hydrophobic interactions and the formation of hydrogen bonds, similar to the inhibition of beta-secretase-1 (BACE-1) enzyme activity [71].

A PLGA-Quercetin NP system was developed, showing a significant role in preventing Aβ fibrillogenesis and the formation of Aβ_42_ peptide aggregates and also being involved in the dissolution of preformed aggregates [72]. Quercetin binds to the nanoparticles and effectively chelates metal ions, thereby preventing the formation of Aβ peptides. These nanoparticles also exhibited reduced cytotoxicity, making them promising candidates for therapeutic applications [72].

Another system of quercetin-modified gold-palladium nanoparticles proved efficient in activating autophagy and promoting the fusion of autophagosomes and lysosomes. Moreover, it can induce Aβ clearance and provide protection against the toxicity resulting from Aβ activity [73].

Quercetin is also under investigation for its potential benefits in ameliorating AD symptoms, with clinical trials currently ongoing. Preliminary findings demonstrated the safety and tolerability of dasatinib and quercetin in a phase I clinical trial [74].

#### 4.1.3. Phytol

Phytol is a natural compound, a part of the chlorophyll molecule, has pharmacological properties, and is used as a precursor to obtain synthetic forms of vitamin E. The study conducted by Sathya et al. [75] on *C. elegans* assessed the potential use of phytol PLGA nanoparticles in the pathology of AD and showed the regulation of AD-associated gene expression, such as ace-1 and hsp-1, thereby reducing Aβ formation. Also, phytol PLGA nanoparticles increased the lifespan of the in vivo models and consequently decreased Aβ deposition and ROS levels [75].

An important aspect of using phytol PLGA NPs is their cholinesterase inhibitory ability. Phytol-encapsulated nanoparticles showed significant inhibition of AChE and the ability to block BuChE activity, highlighting the reduction of AD symptoms [76].

#### 4.1.4. Carotenoids

Another antioxidant used for destabilizing Aβ aggregates is fucoxanthin, a carotenoid obtained from marine brown algae and the most abundant carotenoid found in the marine world. It exhibits important neuroprotective properties, preventing neuroinflammation, oxidative stress, and, most importantly, inhibiting the aggregation of Aβ plaques [77]. Fucoxanthin directly inhibits β-secretase, BACE-1, through interactions between the hydroxyl groups of fucoxanthin and the amino acid residues of BACE-1. Another mechanism of inactivation involves hydrophobic interactions between the amino acid side chains of BACE-1 and the alkenyl chains and geminal dimethyl groups of fucoxanthin. Previous studies have highlighted fucoxanthin’s properties of inhibiting lipopolysaccharide-induced peripheral inflammation through the inhibition of NF-κB and mitogen-activated protein kinase (MAPK) in RAW 264.7 macrophages [78]. An important role of fucoxanthin is its protection against oxidative stress via activation of the PI3-K/Akt cascade and inhibition of the ERK pathway. Neuroprotection in this context is determined by the inhibition of GSK-3β and MEK. An in vitro study conducted by Yang and team [79] using SH-SY5Y cells highlighted the inhibition of Aβ aggregates by PLGA-PEG-Fuc. Besides being incorporated by neurons, the PLGA-PEG-Fuc nanoparticles were incorporated in microglia too, an important aspect for reducing neurotoxicity. Also, this nano-system influenced the Nrf2 and NF-κB signaling pathways, which are essential for regulating oxidative stress and neuronal inflammation [79].

Another important carotenoid used for treating AD pathologies is astaxanthin, a red pigment classified as a xanthophyll and found in a series of microalgae such as *Haematococcus pluvialis*, *Chlorella vulgaris*, *Chlorella zofingiensis*, and *Chlorococcum* sp., serving as an important neuroprotective factor by reducing oxidative stress and promoting neuronal lifespan, particularly for cells located in the hippocampus. A drug delivery system of PEGylated-liposomal astaxanthin was able to recognize Aβ [80], reducing levels of both extra- and intracellular deposition of Aβ, but being more efficient in reducing the heptamer variant of Aβ compared to the monomer variant.

Another significant carotenoid used for treating AD due to its anti-inflammatory and antioxidant activity is lutein. Due to its low bioavailability, pure lutein administration is not as efficient as encapsulating it with a drug delivery system. For the effective delivery and distribution of lutein, researchers explored the use of functionalized cationic biopolymers through the intranasal route for the treatment of AD. The nano-system developed in the study of Dhas and Mehta [81] was composed of a PLGA (poly (lactic-co-glycolic acid)) core, coated with a layer of chitosan, and loaded with lutein. This cationic biopolymer core-shell structure protects lutein from degradation caused by light and high temperatures while enhancing its stability and bioavailability. Their results showed significant nasal mucosal membrane crossing and the system’s capacity to deliver lutein to the brain via the intranasal route, where lutein demonstrated its antioxidant abilities, reducing oxidative stress, which could lead to improved AD treatment outcomes.

#### 4.1.5. Resveratrol

The role of resveratrol in preventing hippocampal neurodegeneration makes this compound suitable for new therapies in treating the symptoms of AD. It has been demonstrated in vitro that resveratrol inhibits β-secretase, thereby reducing the formation of β-amyloid through interaction with the amyloid precursor protein. This inhibitory role is mediated by two trimers, gnetin H and suffruticosol. Administering resveratrol to Aβ PP/PS1 mice for 10 months resulted in improved short-term memory, with a significant increase in the presynaptic protein synaptophysin. Moreover, significant levels of mitochondrial IV complex protein were present, indicating better mitochondrial function and providing evidence of neuroprotection as previously reviewed [82].

Resveratrol is an important antioxidative agent with the notable ability to inhibit phosphodiesterase 4 subtypes and activate the cAMP/PKA pathway. Increased cAMP activity is correlated with lower levels of neuroinflammation and reduced apoptosis. Resveratrol is also responsible for reducing levels of pro-inflammatory cytokines such as IL-1β and IL-6, with an upregulated expression of the antiapoptotic protein Bcl-2 and downregulation of Bax proapoptotic protein expression. An important proof of neuroprotection offered by resveratrol is the elimination of the negative effects of Aβ_42_ on phosphorylated cAMP response element-binding protein (pCREB) and brain-derived neurotrophic factor (BDNF) [82].

A study conducted by Yang et al. [83] characterized a nanoparticle system formed with selenium and resveratrol, which inhibited Cu^2+^-induced Aβ_42_ aggregation by blocking the metal ion binding site on Aβ. Also, the H_2_O_2_ levels induced by the Aβ_42_-Cu^2+^ complex were decreased after the administration of this system [83].

Another study showed the development of a nasal drug delivery system aimed at administering resveratrol more efficiently. In this context, gellan and xanthan gum were used to obtain a nanostructured gel for better and longer adhesion over time, with results showing more effective treatment of AD than orally administered resveratrol suspension [84]. Another intranasal route to bypass the BBB for administering resveratrol is represented by transfersomes coated with gold nanoparticles, which exhibit unique permeation abilities, improving resveratrol cellular uptake and resulting in significant memory recovery [85]. Histopathological examinations on rats showed a significant accumulation of gold nanoparticles in the brain for both transfersomes and nanoemulsions, while transfersomes demonstrated greater accumulation overall [85].

In a study involving 39 patients, it was concluded that a low dose of resveratrol, such as 5 mg, in combination with 5 mg of dextrose and an equal dose of malate or placebo, was tolerable and safe for use over a period of one year, with the treatment administered twice daily [86]. Another study involving a larger group of 119 patients receiving 1 mg of resveratrol or placebo for over a year demonstrated modulation of neuroinflammation and induced adaptive immunity, which are promising for AD therapy [87].

#### 4.1.6. Hesperetin

Hesperetin offers impressive neuroprotective properties, as it can regulate the Nrf2/TLR4/NF-κB pathway, reducing inflammation and acting as an antioxidant agent [87]. Moreover, hesperetin significantly reduced oxidative stress in the hippocampus and cortex of the mice, along with a reduction in lipid peroxidation and ROS levels, as it can upregulate antioxidant genes, such as Nrf2 and HO-1, while downregulating the expression of glial fibrillary acidic protein (GFAP) and Iba-1, responsible for glial cell activation, thus balancing phosphorylated markers such as TLR4 and NF-κB [88]. Hesperetin nanocrystals exhibit great solubility and bioavailability properties, overcoming the low absorption capacity of hesperetin. Nanocrystals shorter than 200 nm have a greater antioxidant capacity due to their kinetic solubility, reducing the levels of free radicals [89].

#### 4.1.7. Ginsenosides

Rg1 is a ginsenoside with multiple mechanisms of action against AD. It regulates various signaling pathways, including PKC, MAPK, PI3K/AKT, and GSK-3β. This ginsenoside ameliorates tau and β-amyloid pathologies by reducing oxidative stress and neuroinflammation, decreasing neuronal apoptosis, and regulating the activity of p38MAPK and PI3K/AKT cellular pathways. Moreover, it stimulates the synthesis of acetylcholine and regulates synaptic function via the BDNF pathway, providing neuroprotective effects and enhancing cognitive performance [90].

### 4.2. Overview of Nanoparticle-Based Drug Delivery Systems

Delivery of antioxidants to the brain presents multiple challenges due to the protective nature of the BBB, restricting the passage of most therapeutic agents. Antioxidants, crucial for combating oxidative stress specific for AD, have significant obstacles in achieving effective concentrations in the brain. In this regard, nanoparticles have emerged as potential delivery systems, providing a promising approach for enhancing drug bioavailability, stability, and targeted delivery. For AD, various types of nanoparticles have been explored, such as polymeric nanoparticles, liposomes, dendrimers, solid lipid nanoparticles, and magnetic and inorganic nanoparticles [91], which will be explored in the following section. Table 1 provides an overview of various recent developments in nanoparticle-based drug delivery systems loaded with natural antioxidants and used in AD treatment.

#### 4.2.1. Polymeric Nanoparticles

The polymeric nature enables precise control over particle stability, controlled release properties, and surface modification capabilities. These nanoparticles can be created from preformed polymers or directly from monomers using various polymerization techniques, and both natural and synthetic polymers can be used for their fabrication. Other methods, such as ionic gelation, solvent diffusion, nanoprecipitation, and spray drying, are also used for polymeric nanoparticle production [109]. Polymers commonly used for delivering therapeutic agents to the central nervous system include polysaccharides, poly (ethylenimines), poly (alkyl cyanoacrylates), poly (methylidene malonates), and polyesters [109].

Polybutylcyanoacrylate (PBCA) nanoparticles coated with polysorbate 80 have been used to deliver small molecules and growth factors for the treatment of AD by using rivastigmine, an acetylcholinesterase inhibitor [110].

Further, poly (lactic-co-glycolic acid) (PLGA) nanoparticles have sustained and controlled drug release, low toxicity, and high biocompatibility, being approved by both the FDA and the European Medicine Agency (EMA) for use in biomedical applications. Mathew et al. used PLGA NPs, modified with Tet1 peptide, to deliver curcumin in GI-1 glioma cell line [67], demonstrating a great potential for AD treatment grace to anti-amyloid and antioxidant properties. Moreover, Fan et al. developed a system of PLGA-PEG NPs functionalized with B6 peptide, a substitute for transferrin, reducing tau phosphorylation and plaque production [66]. Other studies used PLGA NPs for brain delivery of different antioxidants, such as quercetin [72], phytol [75,76], fucoxanthin [79], lutein [81], thymoquinone [104] and ginsenoside Rg3 [105].

Another attractive natural polymer is chitosan, made from chitin, derived from the deacetylation of poly-N-acetylglucosamine. Its muco-adhesion and intrinsic bioactivity can help nanosystems penetrate the brain through the olfactory route and potentially have therapeutic effects against AD, given that chitosan is believed to disrupt intercellular tight junctions, which can increase epithelial permeability [111].

#### 4.2.2. Dendrimers

Dendrimers are a unique type of polymeric nanoparticles known, being highly branched, with well-defined mass, size, shape, and surface properties, used in the diagnosis and treatment of neurodegenerative diseases because they provide sustained therapeutic drug levels, prolonged circulation, and enhanced drug transport and stability. In addition, dendrimers have a strong affinity for proteins, peptides, ligands, lipids, and nucleic acids. There are different types of dendrimers depending on the chemical nature of their core and branches, including polyamidoamine (PAMAM), carbosilane, poly-l-lysine (PLL), and polypropylene-imine (PPI). Among these, PAMAM dendrimers are the most common and are widely used in drug and gene delivery systems, as well as in regenerative medicine [112]. Recently, a less toxic therapy based on tacrine (licensed for AD therapy) and PAMAM dendrimer co-administration was evidenced without modifying the drug activity [113].

#### 4.2.3. Lipid-Based Nanoparticles

Lipid-based nanoparticles have been known for their stability and lack of toxicity, being spherical vesicles made up of one or more layers of phospholipids, the most common types being liposomes and solid lipid nanoparticles (SLNs). Liposomes are particularly interesting due to their ease of preparation, high bioavailability, biocompatibility, and low toxicity, being able to deliver multiple types of drugs: hydrophilic, hydrophobic, and lipophilic. In the treatment of AD, liposomes have shown potential for targeting Aβ peptides and the amyloidogenic pathway [114].

When it comes to the brain delivery of natural antioxidants, many lipid-based nanosystems have been reported. Pinheiro et al. developed two systems for quercetin delivery, one modified with transferrin and one with RVG29 [89,90]. Transferrin-conjugated nanoparticles showed a capacity to inhibit amyloid plaque formation in the hCMEC/D3 cell line, while the lipid nanoparticles modified with RVG29, a peptide that has an affinity for nicotinic acetylcholine receptors, showed a high permeability through the BBB and a capacity to inhibit the formation of Aβ aggregates [93,94]. Moreover, Gu et al. demonstrated the capacity of PEGylated lipid NPs to enhance astaxanthin bioavailability and inhibit plaque formation in APP/PS1 mice [80]. Furthermore, Loureiro and colleagues developed an SLN system for encapsulating resveratrol and grape seed extract that inhibits Aβ aggregation in human brain-like endothelial cells [99]. Santonocito et al. also used stealth SLNs for astaxanthin brain delivery, obtaining 110–150 nm stable NPs with great antioxidant capacity [102].

#### 4.2.4. Inorganic NPs

Inorganic nanoparticles cover a wide variety of compounds, from quantum dots to metallic nanoparticles made of gold, silver, platinum, and iron, as well as magnetic nanoparticles like superparamagnetic iron oxide nanoparticles (SPIONs) or Fe_3_O_4_, being known for their non-toxicity, hydrophilicity, biocompatibility, and exceptional stability compared to organic materials. Studies have investigated gold nanoparticles for their ability to inhibit Aβ fibril formation, tau protein aggregation, neurofibrillary tangle formation, and acetylcholinesterase activity [115]. Liu et al. developed gold-palladium cubic nanoparticles for quercetin delivery, acting on the autophagy-lysosomal pathway in AD, safeguarding SH-SY5Y cells from Aβ-induced cytotoxicity [73]. Also, Salem et al. developed a delivery system based on gold nanoparticles and resveratrol with enhanced permeation through the nasal membrane [85]. Furthermore, mesoporous silica nanoparticles raised as potential drug delivery systems, Halevas et al. developed magnetic core-shell nanoparticles for the delivery of quercetin to the brain [4]. Their system illustrated antioxidant and anti-amyloidogenic properties in primary hippocampal neurons from mice [4].

SPIONs are made from two segments: a magnetic material made of iron and a chemical compartment, being used as theranostic systems in AD. SPIONs nanoparticles with a diameter of 30 to 50 nm have been used for the delivery of quercetin, enhancing neuron survival in AD [98].

#### 4.2.5. Nanoemulsions

Nanoemulsions (NEs) are stable mixtures of nanoscale oil and water phases held in a single phase by surfactant or co-surfactant molecules. They are created through techniques like high-pressure homogenization, ultrasonication, and emulsion inversion point methods. NEs are used to improve drug delivery to specific areas while minimizing adverse effects and toxicity. Additionally, the lipid-like nature of NEs makes them promising for delivering drugs to the brain through the BBB [116]. NEs have been successfully used for the delivery of thymoquinone in high-cholesterol-diet rats, showing a high rate of Aβ plaque clearance [103].

## 5. Discussion

### 5.1. Strengths and Weaknesses of Different Antioxidant-Based Approaches

The bioactive molecules discussed, curcumin, quercetin, phytol, carotenoids, resveratrol, Rg1 ginsenoside, and hesperetin, are known for their potent antioxidant and anti-inflammatory activities, which are crucial in slowing down the progression of AD. They target key pathological features of AD, such as oxidative stress, neuroinflammation, amyloid-beta (Aβ) plaque formation, and cholinergic dysfunction. Each of these molecules acts on distinct but interconnected pathways, as presented earlier, collectively contributing to neuroprotection in AD. Curcumin binds to senile plaques and helps reduce Aβ levels while also activating the Akt phosphorylation pathway and reducing inflammation through the NF-κB pathway [65]. Quercetin [71] and hesperetin [88] target the NF-κB pathway, reducing neuroinflammation and inhibiting acetylcholinesterase to decrease Aβ aggregation, which is crucial for preserving cognitive function, while hesperetin also acts on upregulating antioxidant genes. Phytol addresses cholinergic dysfunction by regulating AchE, thereby improving cognitive outcomes often impaired in AD patients [74]. Carotenoids [78,79,80] exert neuroprotective effects through different mechanisms: fucoxanthin inhibits BACE1, reducing Aβ plaque aggregation; astaxanthin provides general neuroprotection, and lutein specifically mitigates Aβ-induced cognitive deficits. Resveratrol [82] activates the cAMP/PKA pathway and inhibits beta-secretase, reducing neuroinflammation and Aβ plaque formation, addressing both oxidative stress and plaque buildup central to AD progression. On the other hand, Rg1 Ginsenoside [90] regulates the PI3K/Akt and p38MAPK pathways, both involved in modulating neuroinflammation and oxidative stress, highlighting its broad neuroprotective effects.

Despite their promising in vitro efficacy, significant challenges arise when translating these effects to in vivo and clinical contexts. These challenges include poor bioavailability, rapid metabolism, and poor brain penetration, all of which hinder the translation of these bioactive molecules into effective treatments for AD in clinical settings. In order to overcome multiple disadvantages, encapsulating these molecules in nanoparticle systems can enhance their solubility, protect them from degradation, and improve BBB penetration.

Polymeric nanoparticles are widely used due to their versatility, allowing for precise control over particle stability, drug release profiles, and surface modification. This control can be leveraged to improve the bioavailability of therapeutic agents, enhance brain targeting, and provide sustained drug release. Polymers such as PLGA are FDA and EMA-approved, ensuring their safety and compatibility with biological systems. The ability to modify surface characteristics and incorporate targeting ligands enhances the specificity and efficacy of drug delivery to the brain. However, their production processes, such as nanoprecipitation and spray drying, can be complex and require precise control over parameters to ensure consistency [109]. Dendrimers are particularly effective in delivering drugs to the central nervous system (CNS) due to their unique structure, which allows for high drug loading and precise control over drug release. The branched structure of dendrimers allows for a high degree of drug encapsulation, making them efficient carriers for therapeutic agents. They can also be functionalized with various ligands to target specific receptors in the brain, improving therapeutic outcomes. As seen in polymeric NPs, the synthesis and characterization of dendrimers are also more complex, limiting their widespread application.

On the other hand, lipid-based nanoparticles are renowned for their ability to encapsulate both hydrophilic and hydrophobic drugs, making them versatile carriers for AD therapeutics. However, some disadvantages of lipid-based nanoparticles are stability issues, such as leakage and oxidation of the encapsulated drug, which can reduce their effectiveness.

Inorganic nanoparticles, including metallic nanoparticles (e.g., gold, silver, iron oxide), offer exceptional stability and have been explored for both therapeutic and diagnostic purposes in AD treatment. Despite their stability, some inorganic nanoparticles may exhibit cytotoxicity or induce oxidative stress, potentially counteracting their therapeutic benefits. The production and disposal of inorganic nanoparticles, particularly those made from precious metals like gold, can be costly and raise environmental concerns.

Nanoemulsions offer a promising approach for improving drug solubility and bioavailability, particularly for lipophilic drugs, and their lipid-like nature facilitates their transport across the BBB, making them effective for CNS drug delivery. However, nanoemulsions may face challenges related to physical stability, such as phase separation, which can impact their effectiveness. The use of surfactants, essential for stabilizing nanoemulsions, can sometimes lead to toxicity or adverse reactions.

### 5.2. Patient Outcomes and Therapeutic Strategies

While natural antioxidants offer a new approach to treating AD, traditional therapies include cholinesterase inhibitors, such as donepezil, galantamine, and rivastigmine [65], N-methyl-D-aspartate (NMDA) receptor antagonists (memantine), or monoclonal antibodies (lecanemab and donanemab) recently approved after clinical trials [117]. These therapies improve cognitive function by either increasing acetylcholine levels, regulating glutamate activity, or reducing Aβ plaques in the brain. However, their main drawback is their limited impact on the progression of the disease, which only provides symptomatic relief. While these standard treatments are generally accepted for their safety and ability to improve cognitive function, they also have side effects such as nausea and vomiting and concerns about their long-term effects. On the other hand, natural antioxidants are known for their strong antioxidant and anti-inflammatory effects, but their effectiveness is hindered by the need for advanced delivery systems due to poor bioavailability.

When it comes to drug delivery systems, conventional drugs are usually taken orally, which can lead to limited drug penetration across the BBB. Nanoparticle-based systems offer improved drug delivery by crossing the BBB more effectively [111]. However, there are concerns about the long-term toxicity of some types of nanoparticles [91], particularly metal-based systems. Ongoing research is needed to optimize safety profiles and standardization for these systems. Therefore, while traditional therapies are important for managing symptoms, newer approaches like nanoparticle-based delivery systems and natural antioxidants show promise in addressing the underlying causes of AD. However, challenges related to safety, cost, and accessibility need to be addressed to make these strategies more widely available.

Innovative treatments that target various aspects of AD could result in sustained functional abilities in patients (such as eating, dressing, and carrying out day-to-day activities), which are essential for a patient’s mental well-being. Drug delivery systems, such as polymeric NPs, lipidic NPs, inorganic NPs, etc., might enhance these functional outcomes by increasing the bioavailability of drugs, allowing them to penetrate the BBB. Additionally, reducing neuroinflammation with natural antioxidants, like curcumin, could improve overall mobility and physical health by possibly slowing or stopping the progression of motor symptoms, thus enhancing the patient’s welfare.

### 5.3. Challenges and Future Perspectives

Several antioxidant treatments and delivery systems have been proposed as potential interventions for AD. However, it is important to note that many of these approaches are still in the experimental stage and have not been conclusively shown to be effective in AD. Despite their theoretical potential to mitigate oxidative stress and amyloidogenic pathways, the key pathological feature of AD, and the encouraging results observed in early-stage studies, their effectiveness in AD remains unproven, as mentioned by the clinical trials presented earlier [70,74,86,87,118]. Therefore, these approaches should be considered experimental, and further research is essential to establish their clinical relevance in AD.

Moreover, many treatments with positive results in animal or cellular models of AD may not succeed in human trials due to metabolic differences between organisms or contrasting disease pathology. Therefore, future research on antioxidant-based nanoparticle delivery systems should prioritize large-scale clinical trials to verify the potential effectiveness of preclinical therapies.

One major obstacle in developing effective antioxidant therapies is the challenge of achieving adequate bioavailability and targeted delivery to the brain. Many antioxidants suffer from poor absorption, rapid metabolism, and limited ability to cross the BBB. Although current delivery systems, such as nanoparticles and liposomes, are promising, they have not yet been fully optimized or validated for AD. Oxidative stress in AD involves a complex interplay of various ROS, cellular responses, and compensatory mechanisms. Targeting a single pathway or molecule may not be sufficient to achieve therapeutic benefits. Furthermore, the timing of intervention is critical, as oxidative stress plays various roles at different stages of the disease. Understanding the precise mechanisms by which antioxidants can exert neuroprotective effects and determining the optimal time for intervention are ongoing challenges in this field.

In the future, research in the field of AD should prioritize the development of new antioxidant compounds that are more effective at crossing the BBB, have improved stability and bioavailability, and can target the oxidative pathways implicated in AD specifically. Additionally, with the advancement of our understanding of AD pathology and genetics, there is potential for developing personalized antioxidant therapies tailored to individual patients based on their genetic profile, disease stage, and other biomarkers. Combined therapies using antioxidant treatments with other therapeutic compounds, such as tau inhibitors, and the new cutting-edge artificial intelligence tool [118] also hold promise for providing synergistic effects.

## 6. Conclusions

This review provides a comprehensive overview of the latest perspectives on AD treatment, emphasizing the importance of the BBB and the potential of antioxidant-based drug delivery systems. By addressing the challenges of BBB penetration and oxidative stress, researchers aim to develop more effective therapies that can slow or halt the progression of AD and improve the quality of life for patients. Future research directions include investigating novel antioxidant formulations, exploring combination therapies, and integrating advanced nanotechnology approaches to enhance drug delivery to the CNS.

## Figures and Tables

**Table 1:** Nanoparticle delivery systems for natural antioxidants to treat AD.

Antioxidant	Nanoparticle (NP) Type	Targeted Pathway	Mechanism of Action	Advantages	References
Curcumin	PEG-PLA NPs (<80 nm)	-	-	Highly stable Improved bioavailability	[69]
	PLGA NPs modified with Tet1 (150–200 nm)	Oxidative stress response	DPPH radical scavenging activity	Increased neuronal targeting efficiency Antioxidant and anti-amyloid properties	[67]
	PLGA-PEG NPs modified with B6 (<100 nm)	-	-	Biosafety Improved bioavailability	[66]
	Se NPs loaded with curcumin/PLGA composites (160 ± 5 nm)	Amyloidogenic pathway	-	Efficient inhibition of Aβ plaques in transgenic 5XFAD mice	[68]
	Silica NPs (60 nm)	-	-	Biocompatible	[92]
Quercetin	PLGA NPs (<145 nm)	Amyloidogenic pathway	-	Inhibiting Aβ42 fibril formation (with and without Zn2+)	[72]
	Concave cubic AuPd NPs modified with PS-80 (71 nm)	Autophagy-lysosomal pathway	-	High BBB permeability Up-regulating autophagy in SH-SY5Y cell line Clearing Aβ plaques	[73]
	Lipid NPs (SLC and NLC) modified with transferrin (200 nm)	Amyloidogenic pathway	-	NLC inhibit peptide aggregation, but SLC do not Inhibit fibril formation in hCMEC/D3 cell line	[93]
	Lipid NPs (SLC and NLC) modified with RVG29 (<250 nm)	Amyloidogenic pathway	-	Inhibit fibril formation in hCMEC/D3 cell line Increased permeability across in vitro model of BBB	[94]
	Se NPs modified with acacia and PS-80 (89.90 ± 4.17 nm)	Oxidative stress response	DPPH radical scavenging activity	Inhibitory effect on Aβ1–42 aggregation in PC12 cell line	[95]
	Sulfur NPs inserted into microbubbles (<100 nm)	Oxidative stress response	Reduce ER stress and reduce oxidative stress caused by ER stress or eliminate ROS	Reduction of Aβ deposition in the brain of C57BL/6 mice Reduction of inflammation, oxidative stress and nerve cell apoptosis in SH-SY5Y cell line	[96]
	MSNPs modified with PEG3k (200–250 nm)	Amyloidogenic pathway	-	Reduce Aβ aggregation	[4]
	Exosomes NPs (40–150 nm)	Tau phosphorylation	Inhibit CDK5-mediated tau phosphorylation	Inhibit tau phosphorylation in AD mice and reduce formation of NFTs	[97]
	SPIONs (30–50 nm)	Oxidative stress response	Enhance expression levels of antioxidant enzymes and reduces expression of NO synthetase	Better bioavailability Antioxidant effect of QT-SPION in male Wistar rats	[98]
Resveratrol	SLNs modified with OX26 mAb and containing grape seed extract (254 ± 17 nm)	Amyloidogenic pathway	-	Decreased Aβ peptide aggregation	[99]
	SeNPs (<100 nm)	Amyloidogenic pathway	SeNPs have a strong affinity for Aβ42 plaques	Effect on Cu2+-induced Aβ42 aggregation, ROS generation Reduce metal ion induced Aβ42 toxicity in PC12 cell line	[83]
	Nanostructured hydrogel (132 ± 11.90 nm)	-	-	Treats memory loss specific for AD in scopolamine-induced AD in rats	[84]
	Gold NPs (<100 nm)	-	-	Enhanced permeation through the nasal mucosal membrane	[85]
	RBC coated NLC with RVG29 and TPP (<160 nm)	Mitochondrial oxidative stress response	Specifically bind to neurons and target the mitochondria	Improve oxidative stress in APP/PS1 mice Reduce lipid peroxidation damage	[100]
	Selenium/Chitosan NPs (19.93 ± 1.52 nm)	Modulating gut microbiota	Restoring gut microbiota	Inhibit oxidative stress, neuroinflammation, and metabolic disorder in AD mice	[101]
Phytol	PLGA NPs (177.4 ± 5.9 nm)	Mitochondrial oxidative stress response	Stabilizing the mitochondrial membrane	Modulate the expression of apoptotic proteins in transgenic *C. elegans*	[75,76]
Fucoxanthin	PLGA-PEG NPs (<210 nm)	Amyloidogenic pathway	Enhance antioxidant enzyme activity and reduce neuroinflammation	Inhibit Aβ plaques-induced oxidative stress in mice	[79]
Lutein	Chitosan/PLGA SNPs (<150 nm)	Oxidative stress response	Scavenging ROS species	Decrease oxidative stress in SH-SY5Y cell line	[81]
Astaxanthin	SSLNs (< 200 nm)	-	-	Great antioxidant abilities in OEC cell line	[102]
	Liposomal PEG NPs (<80 nm)	Amyloidogenic pathway	Scavenging formaldehyde	Reduce oxidative stress and Aβ plaque aggregation in mice	[80]
Thymoquinone	Nanoemulsion	Amyloidogenic pathway	Modulating APP processing	Reduce Aβ40 and Aβ42 levels in rats Interacts with LRP1, RAGE and BACE1	[103]
	PLGA NPs modified with PS80 (<230 nm)	Oxidative stress response	Reducing superoxide radical production	Improved bioavailability Reduced accumulation of Aβ in albino mice	[104]
Ginsenoside Rg3	PLGA NPs modified with angiopep-2 (100 nm)	Amyloidogenic pathway	Downregulation of AβPP-A4 expression levels	Reduce Aβ plaques in C6 and THP-1 cell lines	[105]
Hesperetin	Nanosuspension	Oxidative stress response	Upregulation of antioxidant enzyme genes	Reduce cerebral oxidative stress in AD mice	[106]
	Nanocrystals	Oxidative stress response	-	Enhance mitochondrial function in SH-SY5Y-APP695 cell line	[107]
	Nanocrystals (170–800 nm)	-	-	Nanocrystals under 200 nm have better antioxidant activity	[89]
Huperzine A	PLGA NPs modified with Lf and TMC (<154 nm)	-	-	Enhanced cellular uptake and distribution in brain using KM mice	[108]
